# Association between serum n-3 polyunsaturated fatty acids and quadriceps weakness immediately after total knee arthroplasty

**DOI:** 10.1371/journal.pone.0228460

**Published:** 2020-01-29

**Authors:** Yusuke Kubo, Shuhei Sugiyama, Rie Takachu, Maki Tanaka, Masae Ikeya, Takeshi Sugiura, Kaori Kobori, Makoto Kobori

**Affiliations:** 1 Department of Rehabilitation, Kobori Orthopaedic Clinic, Nearaichou, Kita-ku, Hamamatsu City, Shizuoka, Japan; 2 Department of Rehabilitation Sciences, Seirei Christopher University, Mikataharachou, Kita-ku, Hamamatsu City, Shizuoka, Japan; 3 Department of Health and Nutrition Sciences, Tokoha University, Miyakodachou, Kita-ku, Hamamatsu City, Shizuoka, Japan; Cleveland Clinic, UNITED STATES

## Abstract

**Objectives:**

Quadriceps weakness (QW) following total knee arthroplasty (TKA) can be elicited by tourniquet-induced ischaemia reperfusion (IR), which causes a vigorous acute inflammatory response. Dietary n-3 polyunsaturated fatty acids (PUFA) are important determinants of organ and tissue protection from IR. This study aimed to examine the association between serum n-3 PUFA levels and QW, knee pain, and knee swelling immediately after TKA.

**Methods:**

A total of 32 patients who underwent unilateral TKA participated in this prospective study. On Postoperative Day 1, serum n-3 PUFA (eicosapentaenoic acid and docosahexaenoic acid) levels were measured. Preoperatively and on Postoperative Day 4, quadriceps strength, knee pain during quadriceps testing, and knee circumference were measured. QW, knee pain, and knee swelling were defined as changes in quadriceps strength, knee pain during quadriceps testing, and knee circumference, respectively, between the preoperative to the postoperative measurement.

**Results:**

Mean serum n-3 PUFA levels were 192 μg/mL (standard deviation, 58 μg/mL) on Postoperative Day 1. All measured variables changed significantly between the preoperative and the postoperative measurement time-points (P <0.01). Quadriceps strength decreased from 1.2 to 0.4 Nm/kg (QW = −65%). Knee pain during quadriceps testing increased from 1.1 to 6.0 (knee pain = 4.0). Knee circumference increased from 40 to 44 cm (knee swelling = 10%). Multivariate analysis showed that lower serum n-3 PUFA levels were independently associated with an increased QW after adjusting for the Kellgren-Lawrence grade and the tourniquet time (P = 0.04). No significant relationship was observed between serum n-3 PUFA levels and knee pain or knee swelling.

**Conclusion:**

Higher serum n-3 PUFA are independently associated with a lower increase in the QW immediately after TKA.

## Introduction

Total knee arthroplasty (TKA) is a common treatment for severe, painful knee osteoarthritis (OA). Although TKA reduces OA-related knee pain, recovery is often compromised by persistent lower extremity muscle weakness, leading to decreased functional performance [1,2]. In the early phase after TKA, quadriceps strength decreases to approximately 80% of its preoperative level [3]. Quadriceps weakness (QW) is associated with decreased gait speed, balance, chair rise and stair-climbing ability, and an increased risk of falls [4–8]. Early postoperative deficits in quadriceps strength can be especially problematic and might exacerbate long-term weakness [9]. Hence, treatment of TKA-induced QW is a significant challenge to improving postoperative recovery.

Previous studies have shown that early QW is associated with knee pain during quadriceps testing, knee swelling, and quadriceps muscle atrophy after TKA [3,10,11]. These factors can be caused by acute inflammation resulting from surgical trauma and/or tourniquet use [12–15]. This study focused on tourniquet-induced acute inflammation, including oxidative stress, which might occur after ischaemia reperfusion (IR) injury [16]. IR injury has been shown to be preventable by nutritional preconditioning [17–19]. Studies have demonstrated that intake of sufficient amounts of dietary n-3 polyunsaturated fatty acids (PUFA), such as eicosapentaenoic acid (EPA) and docosahexaenoic acid (DHA), can limit tissue damage associated with IR [17,18,20,21]. The suspected molecular mechanism underlying this phenomenon can be attributed to the anti-inflammatory and antioxidant properties of n-3 PUFA [22–25]. Dietary n-3 PUFA may contribute to reducing tourniquet-induced acute inflammation in TKA, raising the possibility that n-3 PUFA-based therapy could reduce tourniquet-induced acute inflammation in TKA. As the first step toward assessing the potential benefit of n-3 PUFA-based therapy, this study investigated the association between n-3 PUFA and QW immediately after TKA.

It has been observed that n-3 PUFA metabolites peak approximately 24 hours after IR [26]. In addition, the increased amount of n-3 PUFA after IR is enhanced by n-3 PUFA supplementation taken prior to IR, contributing to the resolution of IR-induced acute inflammation [19]. In the present study, we measured serum n-3 PUFA (EPA + DHA) levels in the morning of Postoperative Day 1, approximately 24 hours after the surgery, to estimate the peak value of n-3 PUFA levels after IR. We hypothesised that the higher serum n-3 PUFA (EPA + DHA) levels, the lower the increase in QW, knee pain, and knee swelling immediately after TKA.

## Materials and methods

### Study design and participants

Eligible patients with OA scheduled for unilateral TKA participated in a single-centre prospective study at an orthopaedic clinic in Japan (Table 1). Data were collected between June 2016 and February 2017. The inclusion criteria were: age 55 to 79 years, body mass index (BMI) 20 to 35 kg/m^2^, ability to understand information provided about the study, and written informed consent. The exclusion criteria were: advanced dementia, serious cardiovascular disease (e.g., requiring warfarin or heparin), diabetes, rheumatoid arthritis, kidney disease, digestive diseases, significant neurologic impairment, inability to perform a muscle strength test due to other diseases or trunk and lower extremity orthopaedic conditions, C-reactive protein level >0.3 mg/dL, taking n-3 PUFA supplements, and cigarette smoking. All patients were fully informed about the purpose of this study and the procedures involved, as well as potential risks associated with participating, and written informed consent was obtained in accordance with the Declaration of Helsinki. This study was approved by the ethical committee of Seirei Christopher University (15093).

**Table 1:** Participant clinical and demographic characteristics.

Characteristic	
Participants, n	32
Age (y), mean ± SD	69 ± 6
Male, n (%)	5 (15)
BMI (kg/m^2^), mean ± SD	26.0 ± 3.2
n-3 PUFA (EPA + DHA, μg/mL), mean ± SD	192 ± 58
KL grade	
Grade 3, n (%)	3 (9)
Grade 4, n (%)	29 (91)
Contralateral Knee	
OA, n (%)	12 (38)
TKA, n (%)	11 (33)
Tourniquet time (min)	59 ± 8

Abbreviations: SD, standard deviation; BMI, body mass index; KL, Kallgren-Lawrence; PUFA, polyunsaturated fatty acids; EPA + DHA, docosahexaenoic acid + eicosapentaenoic acid; OA, osteoarthritis; TKA, total knee arthroplasty

### Perioperative care

All patients were admitted on the day of surgery and discharged home or to a rehabilitation hospital on Postoperative Day 7. Two experienced surgeons performed all surgeries. All patients underwent a tricompartmental uncemented TKA (Low-Contact-Stress implant LCS Complete; DePuy, Johnson & Johnson Co, New Brunswick, NJ, USA) with a medial parapatellar approach, using a pneumatic tourniquet (ATS 2000; Zimmer, Dover, OH, USA), which was applied on the superior aspect of the thigh and inflated to 300 mmHg. Before skin incision, 1,000 mg tranexamic acid was applied topically to the peri-surgical area. A wound drainage system was used and removed approximately 48 hours after surgery. Pain management was performed using spinal anaesthesia supplemented with epidural analgesia peri- and postoperatively for approximately 48 hours, together with a daily maximum dose of loxoprofen (180 mg/d), paracetamol (3 g/d), or celecoxib (400 mg/d) during hospitalisation. When standard pain management was insufficient, flurbiprofen axetil, pentazocine hydrochloride, or diclofenac sodium was used as a rescue analgesic for moderate or severe pain. Postoperative rehabilitation was started in the morning of Postoperative Day 1. All patients were treated two or three times a day (totalling 60–90 minutes per day). The treatment included physical therapy exercise with active and passive range-of motion exercises, strengthening of the quadriceps muscle combined with neuromuscular electrical stimulation, exercise to improve activities of daily living (e.g., the transition exercise from sitting to standing posture, and stair-climbing exercise), walking exercise with a four-wheeled walker from Postoperative Day 1, and walking exercise with T-handle canes from Postoperative Days 4 or 5. If necessary, cold therapy was applied for approximately 10 minutes several times per day.

### Study measurements

All patients were evaluated 1 month before surgery and again on Postoperative Days 1 and 4. On Postoperative Day 1, serum n-3 PUFA (EPA + DHA) levels were measured in the morning. On preoperative 1 month and on Postoperative Day 4, quadriceps strength, knee pain during quadriceps testing, and knee circumference were measured. QW was defined as relative changes in quadriceps strength from the preoperative to the postoperative value. Knee pain was defined as absolute changes in knee pain during quadriceps testing from the preoperative to the postoperative value. Knee swelling was defined as relative changes in knee circumference from the preoperative to the postoperative value. The relative change (%) was calculated using the formula [QW and knee swelling (%) = (postoperative value − preoperative value) / preoperative value × 100]. The absolute change was calculated using the formula (knee pain = postoperative value − preoperative value). All data were collected by four individuals with extensive training in performing measurements. The baseline clinical and demographic characteristics of the study participants, including the Kellgren-Lawrence (KL) grade and the tourniquet time, were obtained from medical records.

### N-3 polyunsaturated fatty acid measurement

Blood samples were collected on the morning of Postoperative Day 1 after a fasting period of at least 12 hours. Measurement of serum EPA and DHA levels was outsourced to Medic (Shizuoka, Japan). In brief, free fatty acids extracted from the serum were analysed by the higher multiple reaction monitoring method using ultra-fast liquid chromatography coupled with tandem mass spectrometry (LCMS-8030, Shimadzu Corporation, Kyoto, Japan).

### Quadriceps strength

Quadriceps strength was measured as the maximal voluntary isometric knee-extension torque using a pull-type hand-held dynamometer (Mobie; Sakai Medical Co., Ltd., Tokyo, Japan). This device consists of a thin pull sensor and a fixation belt. Unlike conventional hand-held dynamometers that measure force by pushing the body against the sensor, knee-extension force can be measured by pulling a distortion gauge associated with a fixation belt. The fixation belt was attached to a leg of the examination couch and to the patient’s ankle at the lateral malleolus. The examination couch was adjusted to the height at which both feet of the patient were just off the floor. Participants were tested in a seated position with a hip angle of approximately 90°, a knee angle of 75° (0° = full extension), while gripping both sides of the couch. Warm-up and familiarisation of this test were performed by asking patients to contract to approximately 50, 80, and 100% of maximal effort. Participants were then instructed to maximally extend their knee against the fixation belt with instructions to “kick as forcefully as possible with a gradual increase in force” for 5 seconds. During each trial, strong verbal encouragement was provided to participants. The testing was repeated up to 3 times with a 1-minute rest interval, and the average of the highest measurement of 2 valid trials was used as the result. Subsequently, quadriceps strength was expressed as the maximal voluntary torque per kilo of body mass using the external lever-arm length and body mass of each participant (Nm/kg). High intra- and inter-examiner reliability of similar quadriceps strength measurements have been reported [27].

### Knee pain

A numeric rating scale (NRS) was used to quantify knee pain during quadriceps testing. Participants rated pain in and around the knee immediately after all measurements using an NRS ruler with a scale from 0 to 10, with 0 representing no pain and 10 representing the worst pain imaginable. The NRS test has shown excellent validity and reliability [28].

### Knee circumference

Knee circumference was measured using a non-stretchable tape measure with the participant relaxed in the supine position. The circumference (to the nearest mm) of the extended surgical knee was measured 1 cm proximal to the base of the patella. The intra-tester and intra-day reliability of this measurement was recently reported to be excellent, with a smallest real difference of 1 cm [29].

### Statistical methods

The Shapiro-Wilk test was used to test the normality of the distributions of the continuous variables. Based on the results, continuous variables were presented as means with standard deviations (SDs), or medians with interquartile ranges (IQRs). Categorical variables were presented as frequencies and percentages. Differences between pre- and postoperative data were examined using paired-samples t-tests or Wilcoxon signed-ranks tests, as appropriate. Univariate and multivariate analyses were used to clarify the association between dependent variables (QW, knee pain, and knee swelling), independent variables (serum n-3 PUFA levels), and possible confounding factors (KL grade and tourniquet time) influencing surgical trauma and IR injury. In these analyses, dependent variables significantly associated with n-3 PUFA on univariate analysis were entered into the multivariate model (Enter method). Subsequently, the multivariate analysis was performed to adjust for potential confounders (KL grade and tourniquet time) in measuring the association between the dependent variables and n-3 PUFA. We conducted all statistical analyses using IBM SPSS version 26.0 (SPSS Inc., Armonk, NY, USA). The threshold for significance was P <0.05.

## Results

The basic characteristics of the study participants are shown in Table 1. The mean n-3 PUFA (serum EPA + DHA levels) on Postoperative Day 1 was 192 (SD, 58) μg/mL.

Table 2 shows the results of the pre- and postoperative measurements. The changes in all measurements were significant (P <0.01). Quadriceps strength decreased from 1.2 to 0.4 Nm/kg (QW, mean increase, 65%); knee pain during quadriceps testing increased from 1.1 to 6.0 (pain intensity; median increase, 4.0); and knee circumference increased from 40 to 44 cm (knee swelling; mean increase, 10%).

**Table 2:** Pre-to postoperative changes in quadriceps strength, knee pain during quadriceps testing, and knee circumference.

Variables	Preoperative	Postoperative Day 4	Change in mean or median from preoperative
Quadriceps strength (Nm/kg) ^‡^	1.2 ± 0.4	0.4 ± 0.2	−65 ± 15 (%)*
Knee pain (NRS)^†^^‡^	1.1 (0, 3)	6.0 (5, 7)	4.0 (2, 6)*
Knee circumference (cm) ^‡^	39.9 ± 4.0	43.9 ± 4.3	10 ± 3 (%)*

Note. N = 32. Pre- and postoperative results presented as mean ± SD, median (IQRs) and P values refer to analyses between pre- and postoperative results using paired-samples t-test or Wilcoxon signed-rank test. Significant changes

* P <0.05.

Abbreviations: Nm, newton meter; NRS, numerical rating scale.

^†^knee pain during quadriceps testing.

^‡^surgical knee.

In the univariate analysis, only QW was found to be significantly associated with n-3 PUFA among the dependent variables (Table 3).

**Table 3:** Correlations and significance between quadriceps weakness, knee pain, knee swelling, n-3 PUFA (EPA + DHA), and confounding factors (KL grade and tourniquet time).

Variables	QW (%)	Knee pain (NRS)	Knee swelling (%)	n-3 PUFA (μg/mL)	KL grade
	r (p)	r (p)	r (p)	r (p)	r (p)
Knee pain (NRS)*	−0.46 (0.01)	―	―	―	―
Knee swelling (%)*	−0.12 (0.52)	−0.15 (0.42)	―	―	―
n-3 PUFA (μg/mL)	0.36 (0.045)	−0.05 (0.80)	0.15 (0.42)	―	―
KL grade	0.21 (0.25)	−0.25 (0.17)	−0.13 (0.47)	0.02 (0.91)	―
Tourniquet time (min)	−0.33 (0.06)	−0.06 (0.73)	0.39 (0.03)	−0.02 (0.94)	−0.06 (0.73)

Note. N = 32. r stands for Pearson or Spearman correlation coefficients. Knee pain was defined as absolute changes in knee pain during quadriceps testing from the preoperative to the postoperative value. Abbreviations: PUFA, polyunsaturated fatty acids; EPA + DHA, eicosapentaenoic acid + docosahexaenoic acid; KL, Kellgren-Lawrence; QW, quadriceps weakness; NRS, numerical rating scale.

*surgical knee.

Multivariate analysis using QW as the dependent variable showed that n-3 PUFA was a significant predictor, after adjusting for confounding factors (KL grade and tourniquet time) (P <0.05, Table 4).

**Table 4:** Multivariate linear regression analysis showing factors that were independently related to quadriceps weakness.

Predictor variable	Standardised β coefficient	95% CI for β		p
		Lower	Upper	
n-3 PUFA (μg/mL)	0.35	0.005	0.17	0.04
KL grade	0.16	−7.1	20.4	0.19
Tourniquet time (min)	−0.32	−1.2	0.03	0.06

Note. N = 32. Adjusted β indicates the adjusted regression coefficient; β stands for regression coefficient. Abbreviation: CI, confidence interval; PUFA, polyunsaturated fatty acids; KL, Kellgren-Lawrence.

This model was statistically stable and able to explain 26% (R^2^) of the variance in change in QW. Multicollinearity was not detected in the multiple regression model (variance inflation factor <2).

## Discussion

To the best of our knowledge, this is the first study to examine the association between n-3 PUFA (serum EPA + DHA levels) and QW, knee pain, or knee swelling immediately after TKA. We found that the higher the serum n-3 PUFA, the lower the increase in QW immediately after TKA, independent of confounding factors (KL grade and tourniquet time). However, no significant association was observed between serum n-3 PUFA and knee pain or knee swelling immediately after TKA.

Some studies in human models have shown that QW is associated with knee pain, knee swelling, and quadriceps muscle atrophy caused by tourniquet-induced acute inflammation in TKA [3,10–15]. Thus, it can be assumed that the stronger the resistance to tourniquet-induced inflammatory response and oxidative stress, knee pain, knee swelling, and quadriceps muscle loss is suppressed, resulting in lower QW. This study focused on n-3 PUFA, which have anti-inflammatory and antioxidant effects on organ and tissue damage caused by IR [17-19,21,24,25,30]. Our study found that n-3 PUFA was an independent predictor of QW immediately after TKA, explaining 26% of variance in increased QW. The mechanism underlying the association between n-3 PUFA and QW immediately after TKA might be the suppression effect of the n-3 PUFA on ischaemic myalgia, muscle swelling, and muscle atrophy in the quadriceps muscle of the knee that had undergone surgery. The synergistic effect of these suppressed factors might contribute to lower QW.

Several animal studies have demonstrated that ischaemic myalgia is driven by IR-induced increase in muscle interleukin-1 beta (IL-1β) and characterised by local mechanical hypersensitivity, decreased muscle strength, and decreased voluntary activity [31,32]. Conversely, n-3 PUFA inhibits the increase in IL-1β after IR injury [33–35]. In the present study, n-3 PUFA seemingly attenuated quadriceps muscle mechanical hypersensitivity through inhibiting IL-1β, increased by tourniquet-induced IR injury. Subsequently, attenuated mechanical hypersensitivity of the quadriceps muscle might have suppressed some of the increase in knee pain during quadriceps testing, indirectly contributing to the decrease in QW. However, our study has found no significant association between n-3 PUFA and knee pain during quadriceps testing (Table 3). This might be due to other factors, including psychosocial variables associated with knee pain immediately after TKA [36], surgical trauma-induced acute inflammation [12], and heterogeneity of postoperative pain management in the amount of prescribed medicine or rescue analgesics. Together, these factors likely resulted in knee pain during quadriceps testing, leading to no significant association between n-3 PUFA and knee pain being observed.

Knee swelling, assessed by knee circumference (1 cm proximal to the base of the patella) using a tape measure, tends to be caused by intra-articular blood accumulation and quadriceps muscle swelling immediately after TKA. The anti-inflammatory properties of n-3 PUFA seem to be effective for quadriceps muscle swelling. A prior study has revealed that n-3 PUFA inhibits microvascular permeability induced by IR in postcapillary venules of the hamster cheek pouch preparation [37]. In the present study, n-3 PUFA might have contributed to decreasing QW through suppression of the quadriceps muscle swelling. However, our study found no significant association between n-3 PUFA and knee swelling, which might be due to the fact that knee swelling reflects intra-articular blood accumulation better than quadriceps muscle swelling. An earlier study has suggested that perioperative blood loss (intra-+ postoperative) is significantly associated with tourniquet time [38]. In our univariate analysis, a significant association was observed between knee swelling and tourniquet time. The mediator of this result may be intra-articular blood accumulation, assuming that all patients have approximately the same amount of drainage volume. In other words, knee swelling may be an indicator of intra-articular blood accumulation. Hence, no significant association was observed between n-3 PUFA and knee swelling.

It has been suggested that muscle atrophy, characterised by activation of cell death and catabolic processes after TKA, is more susceptible to tourniquet use than surgical trauma [13–15], although the muscle atrophy index was not measured in this study. In a previous study, the used magnetic resonance imaging, the quadriceps muscle volume of the operated knee was significantly lower in the group with tourniquet use than that of the non-operated knee. In contrast, in the group without a tourniquet, there was no significant difference in the quadriceps muscle volume pre- and post-operatively [14]. Additionally, previous studies have shown that n-3 PUFA supplementation might attenuate IR-induced cell death in the brain, lung, intestine, and liver [17,19–21]. Furthermore, a recent study in a mouse model of hind limb IR showed that fish oil (n-3 PUFA) pre-treatment suppressed muscle damage through modulating the inflammatory response [30]. In the present study, it is possible that n-3 PUFA contributed to the decrease in QW, through attenuating cell death rate of the quadriceps muscle.

The present study has several limitations. Firstly, QW was assessed only on Postoperative Day 4. The focus of this study, however, was to investigate the effect of n-3 PUFA on QW immediately after TKA. Secondly, some potentially relevant confounders were not accounted for, such as IR-induced quadriceps muscle atrophy and the inflammatory response, which could explain the association between n-3 PUFA and QW immediately after TKA. Future research should pursue a deeper physiological understanding of the association between n-3 PUFA and QW immediately after TKA.

## Conclusions

We found that in patients who had undergone TKA, a higher serum n-3 PUFA was independently associated with lower increase in early QW immediately after TKA. These results suggest the need for an n-3 PUFA supplementation trial for tourniquet-induced acute inflammation in TKA.

## Supporting information

S1 Table n-3PUFA and QW data. Outcomes of the study.
(XLSX)
